# Clustering-Based Multiple Imputation via Gray Relational Analysis for Missing Data and Its Application to Aerospace Field

**DOI:** 10.1155/2013/720392

**Published:** 2013-05-02

**Authors:** Jing Tian, Bing Yu, Dan Yu, Shilong Ma

**Affiliations:** State Key Laboratory of Software Development Environment, Beihang University, No. 37 Xueyuan Road, Haidian District, Beijing 100191, China

## Abstract

A large number of scientific researches and industrial applications commonly suffer from missing data. Some inappropriate techniques of missing value treatment compromise data quality, which detrimentally influences the knowledge discovery. In this paper, we propose a missing data completion method named CBGMI. Firstly, it separates the nonmissing data instances into several clusters by excluding the missing-valued entries. Then, it utilizes the entropy of the proximal category for each incomplete instance in terms of the similarity metric based on gray relational analysis. Experiments on UCI datasets and aerospace datasets demonstrate that the superiority of our algorithm to other approaches on validity.

## 1. Introduction

In a variety of application domains, machine learning and data mining algorithms proved to be of great value [1–3]. However, people using real-world databases or datasets repeatedly encounter the data imperfection issue in the form of incompleteness [4, 5]. Therefore, a plenty of resolutions have been devised to cope with the unfavorable phenomenon. Despite the fact that missing data might not cause any major issue particularly when the missing rate is not significantly high, it could not be considered as an ideal case to ensure the data quality. Additionally, some people argue that the fragmentary data should be excluded from further consideration. Nevertheless, the opinion remains as an obvious shortcoming which is articulated that the other observed factual values of the same instance may simultaneously be absent [6]. In some high-missing-rate environment, this strategy is presumed unreasonable and infeasible. Consequently, the handling for substitution or replacement draws increasing attentions, termed as imputation. In broad outline, the methods available can be separated into two categories: single imputation and multiple imputation methods. On one hand, single imputation, that is, filling in precisely one value for each missing one, intuitively has many appealing features. For example, standard complete-data methods can be applied directly, and the substantial effort required to create imputations needs to be carried out only once [7, 8]. On the other hand, multiple imputation generates a quantity of simulated values for each missing item, in order to reflect properly the uncertainty attached to missing data [9, 10]. This has been advocated as a statistically sound approach, but so far its use has been limited mainly to the social and medical sciences.

Recently, multiple imputation has emerged as an interesting and quite visible alternative in missing data analyses. The versions of the sophisticated approach are advantageous to these conventional techniques because they require less stringent assumptions and mitigate the pitfalls of traditional ones [11, 12]. Nevertheless, for the most part of existing solutions, the following facets retain defective. (a) The clustering strategy combining complete instances with incomplete instances violates the formation of good clusters. In other words, the entire instances involved clustering generates unbiased values due to the imperfection [13]. (b) The traditional *Minkowski*'s *L*_*p*_ (*p* = 1, 2, *∞*) metric is imprecise to scale the similarity among different instances. (c) The current methodologies are hardly applicable to handling the missing aerospace data due to the their underperformance on validity.

In this paper, data imputation is formulated as a problem of estimation of missing values by multiple operations based on clustering. Furthermore, the prime contribution of this paper could be described as follows. (a) Dividing nonmissing items into a finite number of well-partitioned clusters contributes to make the completion in the optimal tailored area. (b) The gray relational analysis, which signifies the situational variation of the curve, could characterize the relative discrepancy more precisely. (c) CBGMI is adopted to the practical aerospace with accurate performance.

The rest of this paper is structured as follows.  Section 2 first briefly introduces both the missingness mechanisms and the emblematic patterns of missing data treatment and then reviews the diverse-related literatures about imputation. In Section 3, the detailed process of the CBGMI algorithm is illustrated in three primary procedural subitems.  Section 4 demonstrates a series of experimental results on both UCI datasets and empirical aerospace datasets to compare the performance with other methods. Finally, conclusions are given in Section 5. 

## 2. Related Work

### 2.1. Missingness Mechanisms

Three different mechanisms, which lead to the introduction of missing values, can be categorized as follows [13, 14].

#### 2.1.1. Missing Completely at Random (MCAR)

When the distribution of an example having a missing value for an attribute does not depend on either the observed data or the missing data. When MCAR happens, evidently the set of subjects with no missing data is also a random sample from the source population. Hence, most simple techniques for handling missing data, including complete and available case analyses, yield unbiased results.

#### 2.1.2. Missing at Random (MAR)

This mechanism establishes once the distribution of an example having a missing value for an attribute depends on the observed data but does not depend on the missing data. Under this scenario, a complete or available case analysis is no longer based on a random sample from the source population, and selection bias likely occurs. Generally, when missing data are MAR, all simple techniques for handling missing data give biased results.

#### 2.1.3. Not Missing at Random (NMAR)

It implies that the pattern of data missingness is nonrandom, and it is not predictable from other variables in the database. If missing data are NMAR, valuable information is lost from the data and there are no universal methods of handling the missing data properly. For instance, participants who are unsatisfied with their company are more likely to not answer the questions about company satisfaction.

### 2.2. Methods for Missing Data Analysis

Current managements of processing missing data can be approximately divided into three categories: tolerable procedures, procedures based on deletion of cases, and imputation-based procedures.

#### 2.2.1. Tolerance

The straightforward method aims to maintain the source entries in the incomplete fashion. Consequently, the ulterior analysis is directly designed based on the raw data [15]. It is poor when the percentage of missing values per attribute varies considerably.

#### 2.2.2. Ignoring

Missing data ignorance often refers to “*Case Deletion*.” It is the most frequently applied procedure, but it is prone to diminish the data quality. Its strength lies in the ease of application; it simply proposes to delete elements with missing data. The procedure can be applied in two manners [12, 16].

- Listwise/Casewise Deletion: it performs indiscriminately deleting from the database any elements with missing data for any of the attributes being examined.
- Pairwise Deletion: incomplete cases are removed on an analysis-by-analysis basis, such that any given case may contribute to some analyses but not to others.

#### 2.2.3. Imputation

*Mean/Mode Substitution* (*MMS*). This is a simple way to impute the missing data. It replaces the missing values by the mean or mode of all the observations or a subgroup at the same variable. It consists of replacing the unknown value for a given attribute by the mean (quantitative attribute) or mode (qualitative attribute) of all known values of that attribute. Replacing all missing records with a single value distorts the input data distribution [15]. 

*Hot-Deck/Cold-Deck Imputation [13]*. Given an incomplete pattern, *hot-deck imputation* (HDI) replaces the missing data with the values from the input vector that is the closest in terms of the attributes that are known in both patterns. This method attempts to preserve the distribution by substituting different observed values for each missing item. Another possibility is the *cold-deck imputation* (CDI) method, which is similar to hot deck, but the data source must be other than the current dataset. For example, in a survey context, the external source can be a previous realization of the same survey.

*Regression Imputation*. This method uses multiple linear regression to obtain estimates of the missing values. It is applied by estimating a regression equation for each variable, using the others as predictors. This solves the problems concerning variance and covariance raised by the previous method but leads to polarization of all the variables if they are not linked in a linear fashion. Possible errors are due to the insertion of highly correlated predictors to estimate the variables. The advantage of this method is that existing relationships between the variables can be used to calculate missing data, but it is rarely used as it amplifies the correlation between variables [13, 16]. 

*Expectation Maximization Estimation* (*EME*). The technique is on the basis of *expectation maximization* (EM) algorithm proposed by *Dempster*,* Laird*, and *Rubin*. The algorithm can handle parameter estimation in the presence of missing data. These methods are generally superior to case deletion methods, because they utilize all the observed data. However, they suffer from a strict assumption of a model distribution for the variables, such as a multivariate normal model, which has a high sensitivity to outliers [2, 14]. 

*Machine Learning-Based Imputation*. It acquires the features of interested unknown data by behavior evolution after sample data processed. The essence is to automatically learn sample for complicated pattern cognition and intelligently predict the missing values. The methods mainly includes decision tree-based imputation, association rules-based imputation, clustering-based imputation, and so forth [6, 8, 17].

*Multiple Imputation*. It replaces each missing value with two or more plausible values that represent the uncertainty about the right value to impute. Each of the two or more resulting complete datasets is then analyzed using standard complete-data methods. All the analyses become combined to reflect both the interimputation variability and intraimputation variability [16, 18, 19].

#### 2.2.4. State-of-the-Art for Missing Data Imputation

Statistical analysis with missing data has been noted in the literature for more than 70 years. Allison [13] pointed that Walks initiated a study on the maximum likelihood estimation for multivariate normal models with fragmentary data. Thereafter, extensive discussions on this topic continue. A useful reference for general parametric statistical inferences with missing data can be found in Little and Rubin [14].

Zhu et al. [19] made use of Magnani's reviewing on the main missing data techniques, including conventional methods, global imputation, local imputation, parameter estimation, and direct management of missing data. They tried to highlight the advantages and disadvantages for all kinds of missing data mechanisms. However, the main problem of these techniques is the need for strong model assumptions. In recent years, many researchers focused on the topic of imputing missing values. S. M. Chen and H. H. Chen [20] developed an estimating null value method, where a fuzzy similarity matrix is used to represent fuzzy relations, and the method is used to deal with one missing value in an attribute. Embedded methods consist of casewise deletion, lazy decision tree, dynamic path generation, and some popular methods such as C4.5 and CART. Nonetheless, these methods are not a completely satisfactory way to handle missing value problems [21]. Firstly, they are merely designed to deal with the discrete values, and the continuous ones are discretized before imputing the missing value, which may lose the true characteristic during the converting process from the continuous value to discretized one. Secondly, they usually studied the problem of missing covariance. 

Huang and Lee [22] employed a grey-based nearest neighbor method to handle the missing data problem. In their opinion, the gray association analysis is employed to determine the nearest neighbors of an instance with missing values. And those unknown values are inferred by the known attribute values derived from these nearest neighbors. Hruschka Jr. et al. [23] used Bayesian networks to fulfill missing values in a hybrid model, which applies the clustering genetic algorithm in objects without missing values and generates Bayesian networks to substitute the missing values. Chen and Huang [24] used the weighted fuzzy rules to estimate null values in relational database. Li et al. [25] borrowed the idea from fuzzy *K*-means clustering and applied it to the problem of missing data imputation with superior performance to the basic *K*-means especially when the percentage of missing values are high. Meesad and Hengpraprohm [26] combined *K*-nearest neighbor-based feature selection and *K*-nearest neighbor-based imputation, including feature selection and estimation of new values. The results showed that the proposed method had powerful estimation ability on microarray datasets. Di Nuovo [2] made the comparisons among four solutions of the *fuzzy c-means* (FCM) in the psychological research environment. The result revealed that the FCM based on optimal completion strategy leads to effective data imputation instead of deleting elements with missing values. Zhang et al. [18] utilized the information within the incomplete instances since the second imputation iteration. The *non-parametric iterative imputation* (NIIA) is an improvement of the classic multiple imputation, which is based on kernel function. The experimental results on UCI datasets unfolded that the NIIA could easily capture the distribution of a dataset even when there is no prior knowledge of the datasets. 

## 3. The CBGMI Algorithm

**Figure 1:**
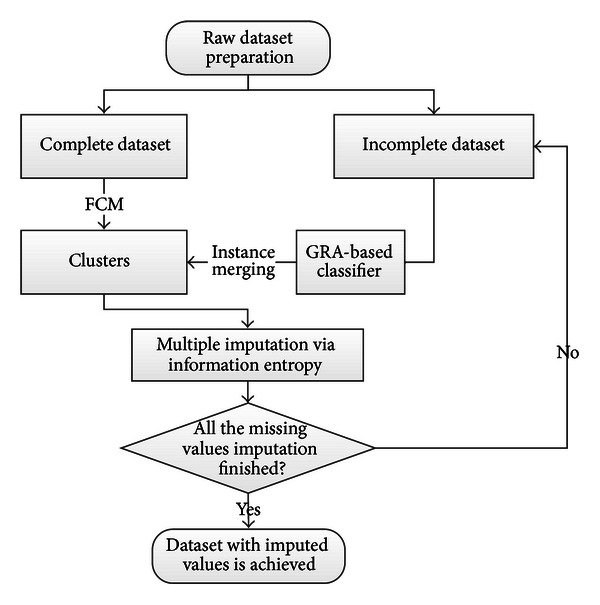
The flowchart of the CBGMI algorithm.

In this section, the global procedure of the CBGMI algorithm is schematized in Figure 1. And each of the key components is detailed correspondingly. Firstly, the clustering technique is explained, and then the computation of gray relational analysis with missing values is presented with formulations. After the instructions of the entropy-based multiple imputation comprising of initial and successive estimations, the entire algorithmic information descriptively listed.

### 3.1. The Clustering Strategy

The specific clustering schema utilizes the standard FCM [1, 27], which attempts to minimize the following objective function with respect to fuzzy memberships *U* ^(*r*)^ = [*u*_*ij*_^(*r*)^] and cluster centroids *C* ^(*r*)^ = *c*_*j*_^(*r*)^: *J* = ∑_*j*=1_^*G*^∑_*i*=1_^*M*^(*u*_*ij*_^(*r*)^)^*s*^ *d*(*x*_*i*_, *c*_*j*_^(*r*)^). In the function, *r* is the ordinal number of the iterations with *x*_*k*_ and *c*_*j*_^(*r*)^, respectively, denoting the *k*th complete data instance and the *j*th cluster, while *d*(·, ·) is the distance metric between two instances, and *u*_*ij*_^(*r*)^ is the degree of membership in which the *i*th instance is subordinate to the *j*th cluster under the “fuzzier” *s*, as *G* defines the total number of clusters, and *M* represents the number of data instances. The algorithm would immediately end with formed clusters under the circumstance that ||*U* ^(*r*)^ − *U* ^(*r*−1)^|| < *ε* or *r* accumulatively reaches the predefined number.

### 3.2. The Classification of Incomplete Instances

Each of the incomplete instances is individually incorporated into the closest cluster according to the maximal value of *gray relational grade* (GRG) [22] in (1) and (2)

(1) GRC(xkmis,ci) =min⁡M min⁡N|xkpmis−cip|+ζmax⁡M max⁡N |xkpmis−cip||xkpmis−cip|+ζmax⁡M max⁡N|xkpmis−cip|     i=1,2,…,M; p=1,2,…,N; 0≤ζ≤1,

where *x*_*k*_^mis^ is the *k*th incomplete instance and *p* is the *p*th attribute with nonmissing values, while *N* is the number of attributes, and coefficient *ζ* is used to decrease the effect of max⁡_*M*_ max⁡_*N*_, which is the maximal value in the matrix.

Consider the following:

(2) $$\begin{array}{c} \text{GRG}\left(x_{k}^{\text{mis}},c_{i}\right)=\frac{1}{N}\sum_{p=1}^{N}\text{GRC}\left(x_{k}^{\text{mis}},c_{i}\right),\;i=1,2,\ldots ,M. \end{array}$$

### 3.3. The Entropy-Based Multiple Imputation

When each time one instance has been assigned to the most proximate group, an internal multiple imputation strategic approach starts as follows.

#### 3.3.1. First Imputation

Algorithms like C4.5 and kNN could be used in the initial round of imputation. Although the MMS was doubted for its potential bias in terms of distributions in the situation, Zhang et al. [18] emphasized that the value of such imputation would be reasonable unless it runs the extraiterative imputation. For this reason, MMS is employed to initialize missing values in the first imputation.

#### 3.3.2. Successive Imputation

*R* = (*r*_*ij*_)_*m*×*n*_ associates with the data matrix of the cluster, into which *x*_*i*_^mis^ ∈ *X*_*ic*_ is attached. That is, it includes *m* − 1 complete elements and one initialized element.

**Step 1**

Calculate the entropy value of the *f*th data instance [3]

(3) $$\begin{array}{c} I_{f}=-k*h_{f}*\ln p_{f},\\ k=\frac{1}{\ln m},\;h_{f}=\frac{||r_{fl}-r_{il}||}{\sum_{i=1}^{m}||r_{fl}-r_{il}||},\;\left(l\neq j\right). \end{array}$$

**Step 2**

Compute the coefficient of difference for the *f*th instance

(4) $$\begin{array}{c} t_{f}=1-I_{f},\;f=1,2,\ldots ,n. \end{array}$$

**Step 3**

Elicit the coefficient of weight for the *f*th copy

(5) $$\begin{array}{c} w_{f}=\frac{t_{f}}{\sum_{f=1}^{n}t_{f}}. \end{array}$$

**Step 4**

Estimate the *j*th attributive missing value of *x*_*i*_^mis^

(6) $$\begin{array}{c} x_{ij}^{\text{mis}}=\sum_{q=1,q\neq j}^{n}w_{q}x_{iq}^{\text{mis}}. \end{array}$$

If the estimated values of the individual instance vary beyond a tolerable interval compared with the calculated value of the last iteration or the number of iteration times does not reach the maximal value, the operations from (3) to (6) continue iteratively. On the contrast, the iterative process mentioned above terminates as the assessed value is considered as the imputed one. Consequently, the imputed instance is aggregated into the corresponding cluster afterwards with updated centroids.

### 3.4. The Framework of the CBGMI Algorithm

 Procedure. CBGMI
 Input. *X*_raw_, the *n* × *m* dimensional dataset with missing values *G*, the number of clusters
 Output. *X*_full_, the *n* × *m* dimensional complete dataset with imputed values
 
*X*_raw_ → *X*_obs_, *X*_mis_, where *X*_raw_ = *X*_obs_ ∪ *X*_mis_ and *∅* = *X*_obs_∩*X*_mis_
 FCM (*X*_obs_, *G*) → *C* = {*C*_1_, *C*_2_,…, *C*_*G*_} according to Section 3.1.

For each element *x*_*k*_ in *X*_mis_

 Allocate *x*_*k*_ to the closest cluster *c*_*q*_ according to Section 3.2.
 Complete the missing values of *x*_*k*_ according to Section 3.3.
 Integrate the *x*_*k*_ into corresponding cluster, and update *c*_*q*_ according to Section 3.3.
  (7) $$\begin{array}{c} X_{\text{full}}⟵\overset{G}{\underset{i=1}{⋃}}c_{i}. \end{array}$$

## 4. Experimental Evaluation

In this section, the assessment criteria are primarily explained in terms of the types of the attributes in Section 4.1. Then, the general effectiveness of our algorithmic approach is presented by a comparative experiment on two UCI datasets [28], remaining superior to MMS, HDI, CDI, C4.5, and EME in Section 4.2.1.  Section 4.2.2 shows the technique which also outperforms these aforementioned approaches by applying CBGMI to a real case analysis in two aerospace datasets.

### 4.1. The Evaluation Criterion

#### 4.1.1. Missing Data on Numeric Attributes

The *root mean square error* (RMSE) is used to evaluate the predictive ability of the various data imputation algorithms within which the attributes are quantitative

(8) $$\begin{array}{c} \text{RMSE}=\sqrt{\frac{1}{m}\sum_{i=1}^{m}{\left(e_{i}-\overset{\sim }{e_{i}}\right)}^{2}}, \end{array}$$

where *e*_*i*_ is the original value, $\overset{\sim }{e_{i}}$ is the predicted plausible value, and *m* is the total number of estimations. The larger value of RMSE suggests the less accuracy that the algorithm holds [18, 29].

#### 4.1.2. Missing Data on Nominal Attributes

The performances of the algorithms for categorical attributes are appraised by the *classification accuracy* (CA)

(9) $$\begin{array}{c} \text{CA}=\frac{1}{n}\sum_{i=1}^{n}l\left({\text{EC}}_{i},{\text{TC}}_{i}\right), \end{array}$$

where EC_*i*_ and TC_*i*_ are the estimated and true class label for the *i*th missing value, respectively, with *n* indicating the total number of the missing values. The function *l*(*x*, *y*) = 1 if *x* = *y*, otherwise *l*(*x*, *y*) = 0. For this reason, the larger value of function *l* indicates the most correct imputed value [18, 29].

### 4.2. Empirical Result Analysis

#### 4.2.1. UCI Datasets

Two datasets from UCI, that is, *Wine* and *Thyroid Disease*, are selected to test the validity of the algorithms. *Wine* contains 178 instances and 13 attributes. The variable values are either real or integer. *Thyroid Disease* includes 7200 instances and 21 attributes. The multivariate factual data are either categorical or real.

To intrinsically examine the effectiveness and validity and ensure the systematic nature of the research, we artificially generated a lack of data at four distinct missing ratios, that is, 5%, 10%, 15%, and 20% under three different modalities, namely, MCAR, MAR, and NMAR in the complete datasets via the means that Twala did [30].

**Figure 2:**
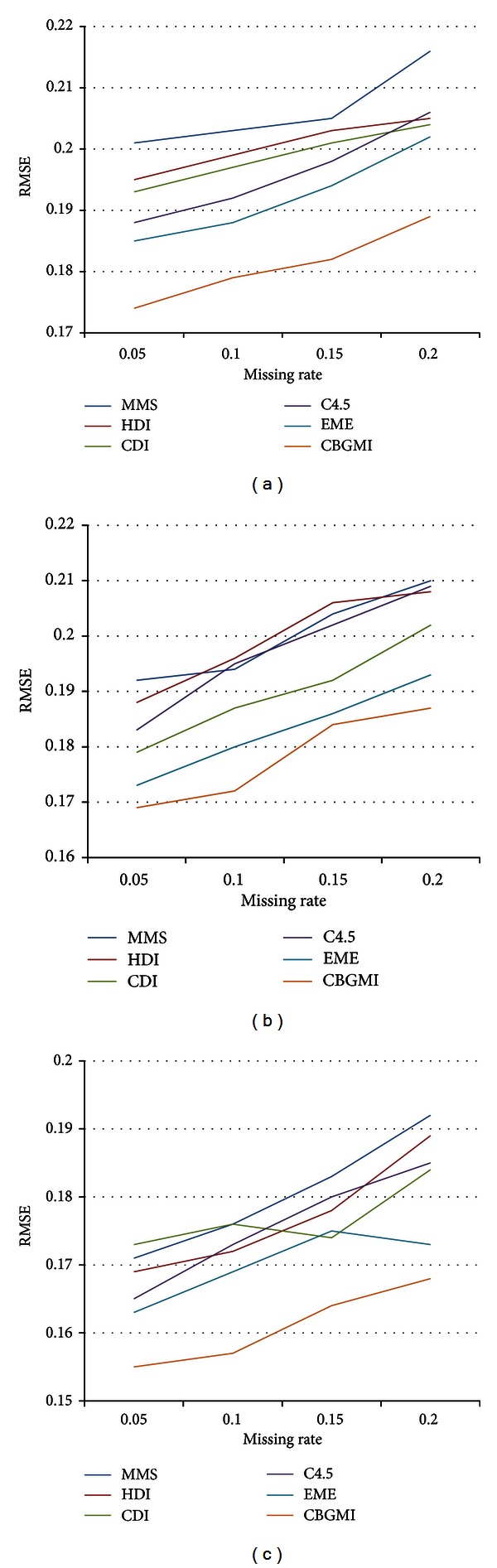
The RMSE in dataset Wine under (a) MCAR, (b) MAR, and (c) NMAR with different missing rates.

**Figure 3:**
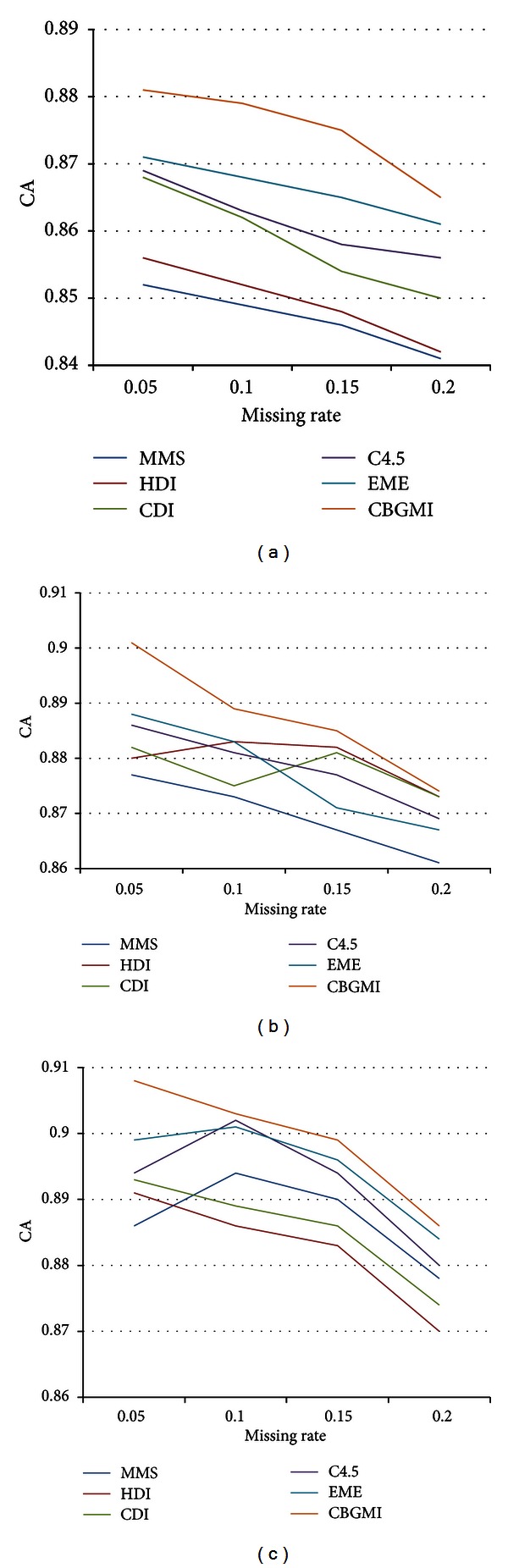
The CA in dataset Thyroid Disease under (a) MCAR, (b) MAR, and (c) NMAR with different missing rates.

As we specify *G* = 3, fuzzier *s* = 1.3, and *ζ* = 0.5, which are mentioned in 3.1 and 3.2, the RMSE produced by six methodologies on the two datasets is illustrated in Figures 2 and 3, respectively.

Figure 2 shows that CBGMI performs better than the other five approaches under all the three missingness mechanisms on Wine, since it is prone to identify the corresponding line from the other five tangled ones. Although the RMSE arises when the missing rate increases from a single subfigure, it indicates that the absence of observed values directly undermines the effect of imputation, as more information could promote the final predictions. Moreover, for each method, the minimum value of RMSE at the same missing ratio always appears when data are NMAR distributed, while MCAR yields the maximum value of RMSE. Therefore, CBGMI is effective and superior to the above algorithms in accuracy for numeric attributes.

The result in Figure 3 demonstrates that CBGMI outperforms the other five approaches under all the three missing modalities on *Thyroid Disease*. The phenomenon that increasing proportion of missing instances deteriorates the CA also states that incomplete nominal values negatively impact on the completion. Similarly, each imputation method functions best under NMAR but operates worst under MCAR. Thus, CBGMI is also applicable to categorical attributes.

To clarify the different distance metrics that influence the accuracy of the results, we suppose that the test happens under MAR. Then, the gray relational analysis metric is practically compared with the *Minkowski distance*, which refers to *Manhattan distance* (MD), *Euclidean distance* (ED), and *Chebyshev distance* (CD).

**Figure 4:**
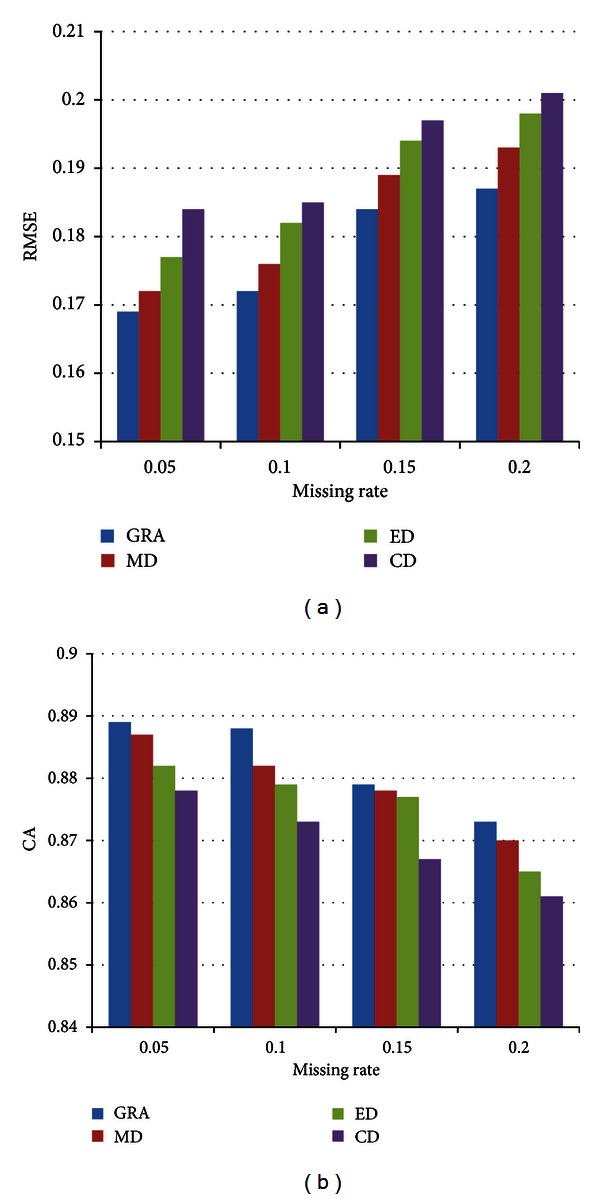
The performances of distance metrics on (a) Wine and (b) Thyroid Disease.

From Figure 4, we could assume that *gray relational analysis*- (GRA-) based distance metric generates the least bias at different missing rates compared with the other three variants of *Minkowski distance*. Furthermore, the discrimination is even more significant when gray relational analysis is contrasted with CD according to RMSE or CA.

#### 4.2.2. Aerospace Datasets

Since Section 4.2.1 testifies the effectiveness of the proposed CBGMI on distinct mechanisms, missing rates, and distance metrics, in this subsection, the missingness is artificially simulated under MAR at missing rate 15% based on gray relational analysis.

To the authors' best knowledge, there are a few of hybrid models integrating multiple imputation into clustering. Accordingly, we selected the method proposed by Zhang et al. [5] (denoted as CGKMI) and replaced our Section 3.3 by NIIA [18] (denoted as CNIIA) as the competitors in this part of experiment.

The *remote controlling for spacecraft flying* (RCSF) dataset covers the data generated by some particular unmanned spaceship on real-time condition when flying in the outer space with the remote controlling by the experts. Due to the huge amount of the raw data, we just extract the data which was produced within one minute. Subsequently, the experiment is designed on the 953 records of 20 continuous attributes.

**Figure 5:**
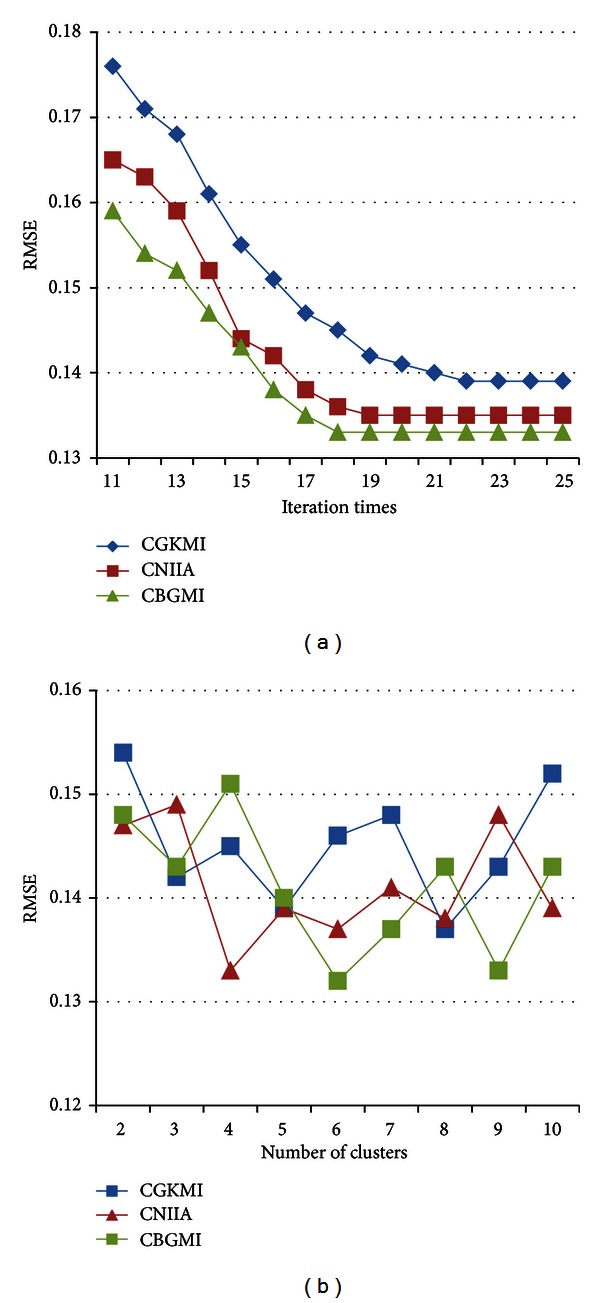
The RMSE influenced by (a) imputation times and (b) number of clusters on RCSF dataset.

When CBGMI is applied to RCSF dataset, the maximum times of the iteration in all the clusters are 18 loops, which is faster than CNIIA's 19 times and CGKMI's 22 times iterations, respectively, in Figure 5(a). What is more, the RMSE achieves slightly lower than the other counterparts.

As versions of clustering principles, interrelationship between RMSE and the number of clusters in these techniques should be discussed. In Figure 5(b), it appears that when the whole data is agglomerated into 6 groups, the RMSE declines to the minimum. Differently, CGKMI performs best with 8 clusters, while CNIIA requires 4 partitions.

The *spacecraft overall mechanical design* (SOMD) dataset comprises the data related to the assembling and fabrication of one specific model of the manned spaceships. Both the numeric values and categorical values are mixed in the dataset. The total number of instances is beyond 300,000. 1,221 elements with the 30 variables belonging to a certain step of the entire manufacturing process are chosen.

**Figure 6:**
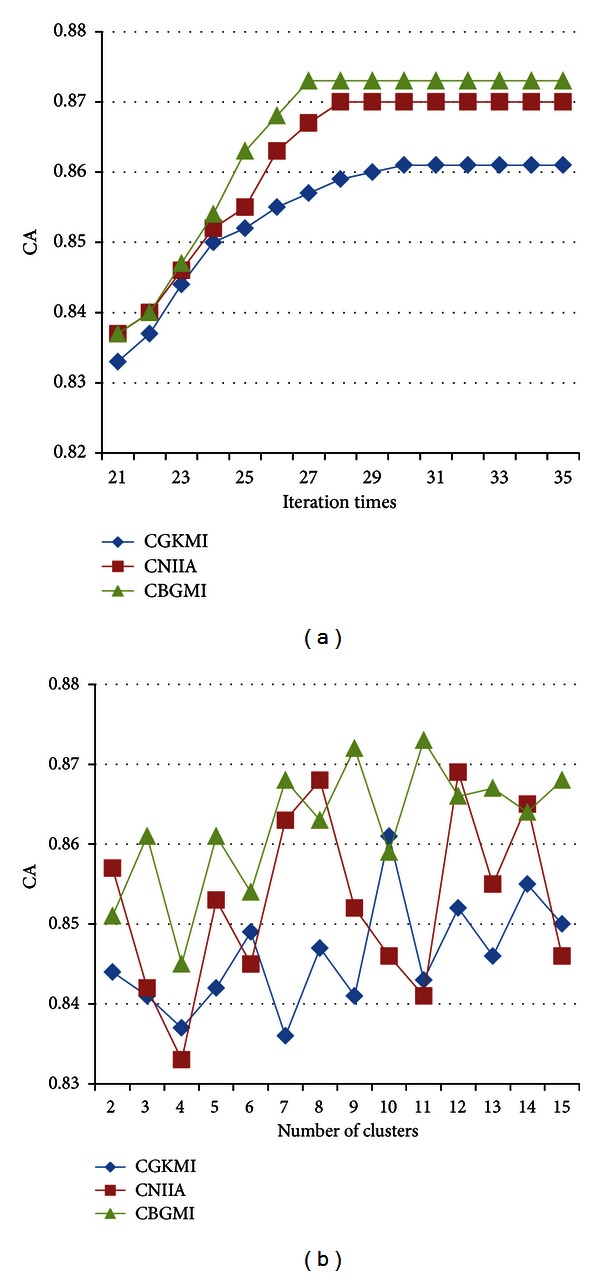
The CA influenced by (a) imputation times and (b) number of clusters on SOMD dataset.

It is easy to perceive that the three algorithms advance CA as the number of iteration aggrandizes until the convergence emerges in Figure 6(a). Simultaneously, CBGMI attains the best CA in the minimum time of the repetitions compared with the other counterparts on SOMD.

When the amount of clusters rises, the CA fluctuates irregularly in the interval (0.83, 0.88). And CBGMI reaches the maximum of CA when 11 clusters exist in Figure 6(b). Generally, CGKMI and CNIIA undulate in an inferior range of CA to CBGMI, which demands the different optimal number of clustered groups, respectively.

## 5. Conclusion

By investigating missing data analysis techniques, this study advocates the clustering-based imputation via partitioning original data into two nonoverlapped subsets, that is, the missing-valued subsets and the complete-valued subsets. Then, the iterative imputation strategy is combined within the categorized groups after each entry including missing values has been merged into the closest cluster through gray relational analysis-based distance metric. The experimental results demonstrate that CBGMI exceeds the existing methods, for example, MMS, HDI, CDI, C4.5, and EME, in terms of the RMSE (for continuous missing attributes) and the CA (for discrete missing attributes) at different missing ratios in two canonical UCI datasets, namely, *Wine* and *Thyroid Disease*. In particular, CBGMI algorithm has been applied into the aerospace datasets. The RMSE and CA affected by the iteration times indicate that CBGMI converges more rapidly than the other iterative imputation techniques with better accuracy in the real application environment. In future research, we will focus on how to more effectively estimate and impute missing values under massive data circumstance.
